# CPD-YOLOv8: A Causality-Driven Physical Modality Fusion Framework for Early Micro-Fire Detection to Protect Complex Wildland Vegetation

**DOI:** 10.3390/plants15192927

**Published:** 2026-09-24

**Authors:** Juan Liu, Ruitong Yao, Qingyuan Yang, Runa Liu, Jie Hu, Jia Lyu, Zichen Wang, Zhilin Zhang, Mengshuai Feng, Feifan Wei

**Affiliations:** 1 Shanxi Agricultural University, Jinzhong 030800, China; 2 Kunming University of Science and Technology, Kunming 650093, China

**Keywords:** vegetation protection, causal inference, precision forestry, physical modality alignment, YOLO architecture

## Abstract

The early detection of wildland forest fires is a crucial task for protecting vulnerable plant ecosystems, yet it remains physically challenging due to the extreme limitations imposed by micro-scale targets and severe environmental interference within dense vegetation. Traditional monocular vision systems typically rely on statistical fitting and are highly prone to false alarms when facing complex optical interference from vegetation canopies, such as penetrating sunlight through foliage and specular reflections. Although multispectral fusion aims to mitigate this issue, existing dual-stream architectures frequently fall into the “heterogeneous negative transfer” trap, where modal conflicts and misaligned physical priors degrade the detection performance. To address these issues, this study introduces a causality-driven and physical-feature-decoupled YOLO (CPD-YOLOv8) framework. Adhering to the principle of physical fidelity, we implement an early R-G-NIR channel replacement strategy to ensure the fundamental physical alignment of spatial and thermodynamic properties. Furthermore, an Adaptive Cross-Modal Attention (ACMA) module is incorporated to facilitate non-destructive feature decoupling, and a Regional Causal-Preserving Downsampling (RCPD) module is designed to intercept environmental confounders (e.g., high-frequency leaf reflections) at the local micro-scale. This framework explores the transition of detection methods from pure statistical correlation toward physical causality. Experimental results on the Corsican wildland multispectral forest fire dataset demonstrate that CPD-YOLOv8 yields an mAP@0.5 of 70.3% and a precision of 87.1%. By mitigating the constraint between precision and recall, this method provides a low-false-alarm solution, offering a technical reference for proactive vegetation management and the reliable deployment of fire early-warning algorithms in real-world complex wildland terrains.

## 1. Introduction

Forest fires represent one of the most severe threats to global plant communities and forest ecosystems. The early detection of micro-fires within complex vegetation environments, particularly in Mediterranean maquis shrublands, is of critical ecological importance. Swift identification at the incipient stage not only minimizes immediate physical destruction, but also plays a vital role in protecting vulnerable flora biodiversity, curbing substantial carbon emissions associated with extensive biomass burning, and preventing the irreversible degradation of fragile plant ecosystems. In regions such as Corsica under a Mediterranean climate, early micro-scale flames can rapidly propagate through dry vegetation under strong winds if not detected in a timely manner, escalating into uncontrollable ecological and economic catastrophes [1,2]. Consequently, employing unmanned aerial vehicles (UAVs) or remote visual surveillance devices for the early detection of micro-scale forest fires has become a vital line of defense for precision forestry and vegetation protection [3].

However, real-world wildland fire surveillance operates under extremely severe physical constraints. On the one hand, early-stage fire targets typically occupy an extraordinarily small pixel proportion in aerial or wide-angle surveillance imagery (often below 0.005%), making their high-frequency edge features highly susceptible to attenuation during deep downsampling processes in convolutional neural networks. On the other hand, wildland environments are frequently subject to severe optical interference from the forest environment, such as heterogeneous light transmission through dense plant canopies, intense solar illumination, specular reflections from wet foliage and rock surfaces, and extreme adverse conditions like heavy smoke occlusion inherent to fires [4]. Traditional visual surveillance relies heavily on monocular visible-light imagery, which frequently encounters severe performance degradation in these extreme environments. Specifically, the RGB modality is highly prone to misinterpreting high-frequency sunlight reflections penetrating the canopy as fire spots, resulting in elevated false alarm rates; concurrently, under dense smoke conditions, it loses the capability to penetrate to the underlying thermal sources, leading to severe missed detections.

Incorporating the near-infrared (NIR) modality—which exhibits strong thermal penetration capability—and synergistically fusing it with RGB features offers an effective pathway to enhance the detection performance in wildland environments. RGB imagery provides fine-grained canopy textures and spatial semantics, whereas NIR imagery directly captures thermal radiation and mitigates illumination interference. However, when dealing with micro-scale targets, existing dual-stream detection networks often compromise the channel priors of source images when relying on straightforward feature concatenation or addition. In complex wildland datasets, such conventional fusion schemes may not only fail to fully exploit the complementary advantages of multiple modalities, but can also trigger heterogeneous negative transfer, ultimately causing model performance to degrade rather than improve. To overcome these limitations, it is imperative to explore a novel network architecture capable of adaptively decoupling features while effectively preserving subtle causal targets.

Existing deep learning object detection models (e.g., the mainstream YOLO series [5,6]) have achieved remarkable performance on standard datasets, but they are susceptible to “statistical fitting” bias in highly complex forest and vegetation environments. Specifically, our preliminary baseline experiments (Exp 3: Native YOLOv8) revealed that while the native monocular model achieved a seemingly acceptable mAP@0.5 of 65.2% on the validation set, this represents, to some extent, a manifestation of “metric deception” [7]. The model tends to overreact to high-frequency interferences, such as strong penetrating sunlight and specular reflections from foliage or wet surfaces. This leads to an elevated false alarm rate, thereby limiting its practical deployment in real-world precision forestry applications.

To mitigate the limitations of monocular vision, most existing multimodal models adopt simple feature concatenation strategies to incorporate infrared thermal imaging data. However, such naive fusion approaches often overlook the issues of physical alignment and semantic gaps between heterogeneous modalities. Our multimodal ablation tests (Exp 11: Early Dual-stream) demonstrate that directly concatenating infrared and visible-light features without adaptive decoupling can trigger modal domain shifts and feature gradient conflicts. This consequently induces the phenomenon of heterogeneous negative transfer, causing a significant drop in detection accuracy (mAP@0.5 decreased to 48.0%) and resulting in missed detections for micro-scale flame targets.

To address the limitations of monocular models and the potential negative transfer in traditional multimodal fusion, we propose a causality-driven and physical modality fusion framework tailored for protecting complex vegetation against micro-scale fires, named CPD-YOLOv8. The overall architecture is illustrated in Figure 1. This study attempts to move beyond conventional architectural stacking strategies, focusing on model optimization from the perspectives of fundamental physical characteristics and causal relationships.

First, we implemented an R-G-NIR channel replacement strategy in the shallow layers of the network, achieving early physical fusion based on thermal radiation. Subsequently, to alleviate the difficulties of coordinating heterogeneous features in deep networks, we designed an Adaptive Cross-Modal Attention (ACMA) module. By leveraging the dynamic weight allocation property of attention mechanisms, this module facilitates the adaptive guidance and decoupling of cross-modal features [8].

Furthermore, to counter the issue that micro-scale forest fires are highly susceptible to losing low-level spatial features in deep networks, we introduced a high-resolution P2 micro-scale detection head. This strengthens the capability of the feature pyramid to capture extremely small targets [9]. Simultaneously, to suppress background artifact interferences (e.g., sunlight reflections on leaves) introduced by shallow high-resolution features, this study introduces the integration of causal inference theory into multimodal vision networks [10]. We designed a Regional Causal-Preserving Downsampling (RCPD) module, which downscales the granularity of causal intervention to the local receptive field region. This module effectively disentangles spurious correlations induced by confounders such as strong reflections, guiding the network to focus more on the thermodynamic causal features of the fire. This promotes a paradigm shift in the detection model from pure “statistical correlation” to integrated “physical causality”.

Based on multiple rounds of architectural deduction and ablation analysis, we conducted a systematic study on the core challenges of wildland micro-scale forest fire detection. The main contributions of this paper can be summarized as follows:

- In-depth analysis of information attenuation in micro-scale features during the downsampling process. We observed that under the wide-angle field of view of UAVs monitoring plant canopies, traditional Intersection over Union (IoU)-based loss functions exhibit high sensitivity to extremely small targets, which can compromise the stable convergence of the model [11]. Furthermore, from the perspective of feature evolution, conventional global pooling operations in shallow, high-resolution networks cause significant information loss for the high-frequency features of micro-scale flames. This provides theoretical support for subsequently optimizing pooling operations and shifting toward local feature preservation strategies.
- Exploration of the applicability of multimodal physical feature alignment in data augmentation. Through ablation experiments, this study found that when processing dual-stream networks, an over-reliance on conventional image augmentation techniques (e.g., MixUp [12] or high-intensity CLAHE) may, to some extent, weaken the physical correspondence between thermal radiation and visible light in the low-level spatial domain. This subsequently impairs the effectiveness of multimodal fusion and causal inference. Consequently, we propose that in the data preprocessing of complex forestry multimodal scenarios, “physical feature fidelity” should be appropriately prioritized.
- Introduction of a causal decoupling mechanism to alleviate the constraint between precision and recall. Addressing the limitation that a single mAP evaluation metric struggles to comprehensively reflect false alarm conditions in practical disaster early-warning systems, this study draws inspiration from causal inference theory [13] to perform feature filtering via the RCPD module and the cross-modal attention mechanism (ACMA) [14]. This mechanism effectively suppresses environmental confounders such as strong sunlight reflections in natural environments. While maintaining a reasonable recall rate, it successfully controls the false alarm rate of the model, providing a reliable improvement approach for micro-scale target detection in complex vegetation environments.

## 2. Related Work

### 2.1. Object Detection in Complex Wildland Environments

In recent years, deep learning-based object detection technologies have made remarkable progress. In particular, the YOLO series and its derivative frameworks [15,16,17] have demonstrated a favorable trade-off between accuracy and real-time performance on benchmark datasets. To extend these algorithms to practical engineering applications, numerous researchers have incorporated Feature Pyramid Networks (FPN) [9] or various attention mechanisms to enhance model robustness against complex background environments.

However, most existing models operate under the implicit assumption that targets possess relatively complete spatial contours and moderate pixel proportions. When applied to large-scale, complex wildland surveillance (e.g., UAV-based forest fire patrols), early flames typically occupy an extremely low pixel proportion (i.e., micro-scale targets) and are frequently accompanied by severe natural interferences, such as dynamic illumination variations and specular reflections from water or rock surfaces. Under these challenging visual conditions, conventional deep convolutional downsampling processes inevitably induce irreversible information attenuation of micro-target features. Moreover, models are prone to misclassifying high-brightness background noise as genuine fire sources. Consequently, effectively suppressing wildland background artifacts while simultaneously preserving micro-scale spatial features remains a critical challenge.

### 2.2. Multi-Modal Fusion Strategies (RGB & Thermal/NIR)

Given the inherent limitations of visible-light imagery under low-illumination or dense smoke occlusion conditions, multimodal detection incorporating thermal infrared imaging has increasingly emerged as a research hotspot [18]. Mainstream multimodal fusion paradigms primarily encompass early fusion, feature-level fusion, and decision-level fusion. Among these, feature-level dual-stream networks typically employ feature concatenation or cross-modal attention mechanisms to integrate heterogeneous features [19].

Nevertheless, we observe that such fusion strategies based on conventional statistical distributions still face distinct challenges in wildland micro-scale fire detection. Fundamentally, infrared and visible-light modalities differ in their underlying physical dimensions; naive feature stacking can induce feature conflicts, resulting in a partial loss of pre-trained prior knowledge [20]. Furthermore, subtle spatial parallax between heterogeneous sensors can adversely affect fragile thermal radiation features during strict alignment. Therefore, exploring mechanisms that can maintain thermodynamic independence while achieving soft cross-modal decoupling is critical for highly robust patrol systems.

To address the aforementioned challenges, recent literature has explored advanced modulation and interaction paradigms for integrating complementary features across various remote sensing domains. For instance, HucrTrack [21] employs dual-representation interaction to achieve unified contextual reasoning in hyperspectral tracking; CASS [22] utilizes spectral-spatial modulation to process complex multispectral inputs from UAVs; and STAR [23] introduces a unified spatiotemporal fusion framework for satellite video object tracking. These state-of-the-art frameworks provide profound architectural context for bridging modality gaps through sophisticated cross-modal interactions. Inspired by these structural insights, yet focusing on the unique physical dimension gaps inherent in wildland micro-scale fire detection, our proposed ACMA specifically introduces a cross-modal gating mechanism. Unlike general spatiotemporal or spectral modulations, this gating strategy achieves targeted soft decoupling, effectively preserving independent thermal radiation signatures while dynamically suppressing vegetative background confounders.

### 2.3. Causal Inference in Computer Vision

As deep models increasingly suffer from spurious correlations inherent in data distributions, causal inference theory has been introduced into the computer vision domain [13]. Existing visual causal studies predominantly employ techniques such as backdoor adjustment to block the impact of environmental confounders, thereby guiding networks to learn intrinsic target features [10,24,25,26].

Although global causal interventions have proven effective in macro-scale scenarios, their application to wildland micro-target detection presents distinct obstacles. When a flame target occupies merely a few dozen pixels, confounder representations extracted via global pooling are easily diluted by vast background vegetation. As a result, localized high-frequency interferences (e.g., intense leaf reflections) become smoothed, rendering causal gating mechanisms ineffective at precise capture. Consequently, investigating strategies to downscale the granularity of causal intervention to local micro-regions—thereby achieving fine-grained decoupling of local confounders—represents a highly valuable topic in current causal representation learning.

## 3. Methodology

The core objective of our research was to achieve the precise detection of early-stage fire targets with extremely low pixel proportions (typically <0.005%) under extreme wildland physical environments. Traditional models applied to this task are susceptible to the dual impacts of “feature attenuation” and “environmental spurious correlations”. To address this, this paper proposes a framework combining causality-driven mechanisms with physical modality fusion (CPD-YOLOv8).

### 3.1. Re-Evaluating the Modality Fusion Pipeline

#### 3.1.1. Exploring the Boundaries of Statistical Augmentation: The Underlying Logic of Physical Spatial Alignment

Mainstream models rely heavily on statistical data augmentation (e.g., MixUp [12] and Contrast Limited Adaptive Histogram Equalization, CLAHE) to prevent overfitting. However, this study observes that augmentations based solely on the visible-light domain may, to a certain extent, disrupt the physical correspondence of multimodal data. For instance, superimposing infrared pixels onto the background in a semi-transparent manner may distort the thermal radiation priors; conversely, excessively enhancing contrast amplifies high-frequency background noise, interfering with subsequent feature decoupling.

In the data processing stage, rather than simply concatenating the RGB and NIR modalities (captured within the 750–1000 nm spectral band) into a four-channel tensor, we adopted a channel-substitution strategy by replacing the blue (B) channel with the NIR thermal channel. This design is motivated by two critical considerations. First, from a computational architecture perspective, maintaining a three-channel input preserves the structural priors of the ImageNet pre-trained weights, avoiding the convergence instability and additional parameter overhead associated with modifying the first convolutional layer for four channels. Second, from an ecological optics perspective, blue light possesses shorter wavelengths and is thus highly susceptible to Rayleigh scattering within dense plant canopies and atmospheric haze. By discarding the high-frequency noise of the B channel and replacing it with the NIR channel—which exhibits superior penetrability and sensitivity to micro-heat sources—our model effectively mitigates the false alarms triggered by vegetation reflections while perfectly retaining the structural contours provided by the red and green channels.

Based on these observations, our architecture favors a preprocessing approach prioritizing “physical fidelity”. We implemented an early channel replacement strategy by embedding the infrared image into the blue (B) channel of the visible-light image. This constructs a pseudo-color R-G-NIR tensor, aiming to fundamentally promote the spatio-temporal alignment of thermal radiation priors with visible-light spatial contours.

#### 3.1.2. Heterogeneous Modality Conflicts and the Construction of an Asymmetric Dual-Stream Backbone

Due to the inherent discrepancies in the physical properties of visible and infrared light, directly utilizing a single-stream network to extract features from the R-G-NIR tensor may induce domain shifts. To mitigate this, we designed an asymmetric dual-stream architecture. In the shallow layers, the network decouples the tensor once again, routing it into the visible-light backbone and the infrared backbone, respectively. To achieve deep adaptive interaction, we designed the Adaptive Cross-Modal Attention (ACMA) module, the detailed structure of which is illustrated in Figure 2.

During the process of feature alignment and weighting, the ACMA module dynamically generates attention masks via a cross-modal guidance mechanism. Let $F_{vis}\in R^{C\times H\times W}$ denote the visible-light branch features and $F_{ir}\in R^{C\times H\times W}$ denote the infrared branch features, where C, H, and W represent the channel depth, height, and width, respectively. Both undergo linear mapping to obtain Queries (Q), Keys (K), and Values (V).

The cross-modal weights are calculated as follows:

(1) $$Q_{vis}=\sigma (W_{q}^{vis}*F_{vis}),K_{ir}=\sigma (W_{k}^{ir}*F_{ir})\;$$

(2) $$A_{vis\to ir}=\sigma (W_{a}^{ir}*K_{ir}),A_{ir\to vis}=\sigma (W_{a}^{vis}*Q_{vis})\;$$

where ∗ denotes spatial convolution with 1×1 projection kernels, and σ (⋅) represents the Sigmoid activation function. The final fused feature $F_{fused}$ is concatenated from the bidirectionally enhanced features:

(3) $$F_{fused}=\delta (BN(W_{f}*Concat(V_{vis}⊙A_{vis\to ir},\;V_{ir}⊙A_{ir\to vis})))$$

Here, $V_{vis}\in R^{C\times H\times W}$ and $V_{ir}\in R^{C\times H\times W}$ represent the explicit Value tensors for the visible and infrared modalities (where $V_{vis}=F_{vis}$ and $V_{ir}=F_{ir}$ in this architecture). Physically, $V_{vis}$ encapsulates high-resolution visual details such as smoke texture, color distributions, and plant canopy boundaries. In contrast, $V_{ir}$ encodes thermal radiation magnitude and localized heat intensity capable of penetrating dense smoke cover. During feature fusion, $V_{vis}$ and $V_{ir}$ are gated by cross-modal attention maps via element-wise multiplication (⊙) and concatenated along the channel dimension, resulting in a joint tensor of shape $R^{2C\times H\times W}$. A subsequent 1×1 convolution $W_{f}$ reduces the dimension back to $R^{C\times H\times W}$, followed by Batch Normalization (BN) and SiLU activation ( δ ).

To alleviate the impact of initial random noise in the infrared branch on the visible-light priors, this model employs a “two-stage freeze-and-wake” strategy: the deep weights of the visible-light backbone are first frozen to warm up the ACMA; once the infrared features stabilize, the entire network is awakened (unfrozen) for fine-tuning.

### 3.2. Micro-Scale Representation and the Pooling Paradox

#### 3.2.1. High-Resolution P2 Detection Head for Micro-Scale Targets

Conventional models typically output detection results at 8×, 16×, and 32× downsampling stages, during which the spatial features of micro-scale forest fires are highly susceptible to being smoothed out. To mitigate this issue, we extracted high-resolution feature maps from the shallowest layer of the backbone network (at merely 4× downsampling) [9] to construct a P2 micro-scale detection head, aiming to capture the edge features of flames more distinctly.

#### 3.2.2. Analysis of Information Attenuation in Micro-Scale Features During Pooling Operations

The P2 layer inevitably retains a portion of background artifacts. In the attempt to suppress these noises, this study analyzed the behavior of micro-scale features during pooling operations: traditional IoU-based loss functions (e.g., GIoU [11], DIoU [27]) are prone to inducing gradient fluctuations when targets are extremely small; simultaneously, the receptive field of conventional noise reduction operations (such as global average pooling) is vastly larger than the target scale, easily causing the activation values of the micro-flames to be smoothed and diluted by the surrounding dark background pixels. Consequently, it is imperative to explore more fine-grained, localized feature intervention strategies.

### 3.3. Causal Intervention Modules (CPD & RCPD)

To alleviate the “precision–recall trade-off” effect introduced by the P2 layer, this study introduces causal inference theory [13], attempting to suppress environmental spurious correlations from the perspective of data generation mechanisms.

#### 3.3.1. Causal Graph Construction in Complex Wildland Interference Scenarios

We constructed a structural causal model suitable for forest fire detection. Let X be the image feature, Y be the detection result, and C be the environmental confounder (e.g., intense sunlight, high-frequency specular reflections from water surfaces). The network is prone to establishing a spurious correlation path X←C→Y. We need to utilize backdoor adjustment to block the backdoor path from C to X and calculate the interventional distribution P(Y|do(X)) [10].

According to the backdoor criterion, the post-intervention probability can be expressed as:

(4) $$P(Y|do(X))=\sum_{z\in Z}P(Y|X,z)P(z)$$

where Z represents the discretized dictionary of the confounder set. In practice, since the true global distribution of environmental confounders in complex wildland scenarios is unobservable and computationally intractable, the Regional Causal-Preserving Downsampling (RCPD) module approximates the prior probability P(z) using spatial priors within a local receptive field. Specifically, we estimate P(z) by computing the spatial mean (e.g., via average pooling) over local patches, which acts as a dynamic surrogate for the regional background confounder distribution. Compared to standard local attention mechanisms that merely re-weight features based on observational correlations—which often inadvertently amplify the spurious correlation path X←C→Y—our causal intervention explicitly adjusts for these confounders. By stratifying the local background context through the approximated P(z), the RCPD module inherently decouples genuine fire features from environmental noise, ensuring that the model relies on true causal semantics rather than deceptive background anomalies.

#### 3.3.2. Localized Downscaling Mechanism of the Regional Causal-Preserving Module (RCPD)

Based on the backdoor adjustment theorem, conventional causal intervention modules (CPD) typically extract global feature distributions [28] to simulate P(Y|do(X)). However, when dealing with micro-scale targets, a global receptive field tends to average out the influence of local confounders. To address this, this paper designed a Regional Causal-Preserving Downsampling (RCPD) module, as illustrated in Figure 3.

RCPD downscales the granularity of causal intervention into a 5×5 local sliding window. Within each local micro-region, it independently constructs a confounder dictionary and calculates causal gating weights. This mechanism attempts to achieve the fine-grained decoupling of local high-frequency reflections. For micro-scale targets, an appropriately reduced causal decoupling receptive field can strike a favorable balance between suppressing local noise and preserving weak features.

## 4. Experimental Setup

To comprehensively evaluate the performance of the CPD-YOLOv8 architecture in extreme physical environments, this section details the dataset construction, evaluation metrics, and underlying implementation details of the experiments.

### 4.1. Datasets and Metric Constraints

This study utilized a multispectral forest fire dataset constructed based on the real-world wildland environment of Corsica, aiming to compensate for the inadequacies of certain traditional public datasets (e.g., BoWFire [29], FLAME [30]) in large-scale complex terrains and multi-interference environments.

Our team’s selection of Corsica Island as the target detection region for this study was driven by profound geographical and physical considerations. As illustrated in Figure 4, the environmental characteristics of this region constitute typical “natural catalysts” that trigger the escalation of micro-fires into uncontrollable disasters. Secondly, the island is widely covered with dense Mediterranean maquis shrublands. Rather than merely treating this continuous biomass as an abundant fuel base, it is crucial to recognize it as a vulnerable plant ecosystem rich in endemic flora. Early fire monitoring here is not just about disaster control, but represents a preemptive measure to protect these fragile botanical species from irreversible ecological degradation. In these real-world wildland environments, the highly heterogeneous canopy structures, coupled with the seasonal discoloration of dry foliage, create exceptionally complex background patterns. These natural textures closely resemble the visual features of early-stage smoke and micro-flames, significantly increasing the risk of false negatives in visual monitoring. Finally, the rugged and complex mountainous terrain of Corsica not only easily generates channeling effects that amplify wind speeds and exacerbate rapid fire spread, but also introduces severe visual occlusions and dynamic lighting variations for UAV visual patrols. Therefore, a dataset constructed based on this high-risk zone can most authentically replicate the severe challenges faced in wildland surveillance, such as complex backgrounds and micro-scale targets.

#### 4.1.1. Hardware Consistency and Scale Challenges Induced by Variable Altitudes/Angles

Distinct from datasets collected at fixed altitudes and single perspectives, the dataset utilized in this study captures spontaneous, real-world wildland fires rather than artificial controlled burns. Although captured by the same device (UAV), the collection process spanned various flight altitudes and highly variable gimbal pitch angles. This collection strategy strictly mirrors actual wildland patrol operations, resulting in significant variances in the Ground Sample Distance (GSD) of targets within the frame. Notably, some early-stage forest fire targets exhibit extreme micro-scale characteristics (pixel proportion < 0.005%). Concurrently, the dataset realistically encompasses complex wildland scenarios such as penetrating sunlight, specular reflections from water and rock surfaces, low illumination, and dense smoke occlusion.

Considering the potential data limitations of infrared sensors in practical patrols, we introduced an “asymmetric hybrid construction strategy”:

- Multimodal Core Aligned Subset: 635 pairs of spatially and temporally aligned visible-light and infrared images, designed to explore the potential of cross-modal feature fusion.
- Monocular Robustness Testing Subset: 500 complex, pure visible-light images (without infrared assistance), aimed at validating whether the model could maintain relatively stable monocular detection robustness in the absence of infrared inputs.
- To comprehensively evaluate the proposed model, the constructed dual-modal dataset, comprising 635 paired images, was randomly partitioned into training, validation, and testing sets at a ratio of 8:1:1 (i.e., 507, 64, and 64 pairs, respectively). Within this multimodal framework, the RGB images primarily provide fine-grained visual details, including the spatial textures and color distributions of forest backgrounds and smoke. In contrast, the complementary infrared images capture critical thermal signatures, enabling the model to bypass visual confounders. This dual-modality configuration ensures that the network learns robust, causal features rather than relying on spurious environmental correlations.
- To strictly prevent data leakage and rigorously evaluate the model’s generalization capabilities, the dataset partitioning strictly followed a sequence-disjoint (or scene-disjoint) protocol rather than naive frame-level random shuffling. Specifically, frames extracted from the same continuous UAV flight sequence or capturing an identical geographic fire scene were strictly isolated into the same subset (i.e., exclusively in the training, validation, or testing set). This strategy ensures that the model cannot trivially achieve high performance by memorizing highly similar background features from adjacent temporal frames, thereby proving its robustness in unseen environments.

#### 4.1.2. Preprocessing Strategies for Physical Alignment and Statistical Augmentation

Traditional object detection research typically relies on augmentation techniques like MixUp to suppress overfitting [31]. However, as previously discussed, in multispectral causal detection tasks, augmentations based on pixel mixing may interfere with cross-modal spatial and thermodynamic alignment. Excessive application of adaptive histogram equalization may also amplify the high-frequency noise floor. Therefore, during the training process of this experiment, strict limitations were imposed on strong statistical augmentation strategies that could significantly alter the fundamental physical properties of the images.

### 4.2. Evaluation Metrics

In wildland surveillance scenarios, a traditional single-metric evaluation system (e.g., mAP@0.5) may struggle to comprehensively reflect the model’s performance in practical deployment [32,33]. A model might inflate its recall and mAP scores by generating broad responses to high-frequency regions.

However, in real-world deployment, frequent false alarms severely diminish the system’s usability, while missed detections introduce safety hazards. Therefore, in addition to adopting the standard mAP@0.5, this study places greater emphasis on precision performance. When addressing the precision–recall trade-off, this research leans toward suppressing obvious background interferences, aiming to achieve a lower false alarm rate while maintaining a reasonable recall rate, thereby aligning more closely with the demands of reliable practical deployment.

### 4.3. Implementation Details

The hardware configuration employed an NVIDIA RTX 4090 GPU (24 GB VRAM) and an Intel Core i9 processor, operating on the PyTorch 2.1 framework. For the “two-stage freeze-and-wake” training strategy of the dual-stream network:

- Stage 1 (Frozen Warm-up Period): The visible-light backbone (Layers 0–9) was frozen, and HSV color space augmentation was disabled. The initial learning rate was set to 0.001, utilizing a smaller batch size to stabilize gradients during training for 100 epochs.
- Stage 2 (Full-Network Awakened Fine-Tuning Period): The entire network was unfrozen, and the learning rate was reduced to 0.0001. Fine-tuning was conducted for 200 epochs using the SGD optimizer coupled with a cosine annealing decay strategy. All input images were uniformly resized to a resolution of 640 × 640.

## 5. Experimental Results and Ablation Study

This section progressively investigates the effectiveness of the CPD-YOLOv8 architecture through ablation experiments and qualitative analysis. We detail the evolutionary process from the baseline model to the optimized architecture and analyze the impact of each module on the overall performance.

To clearly illustrate the evolutionary trajectory of the model architecture optimization, Table 1 summarizes the four core stages that we underwent during the ablation study and their corresponding key observations. This facilitates an understanding of the model’s comprehensive path from initially encountering physical and cognitive bottlenecks to ultimately achieving a performance breakthrough.

Building upon this, we conducted extensive quantitative evaluations. Table 2 details the specific performance metric results under different configurations, further substantiating the quantitative basis for the aforementioned phase transitions.

To rigorously validate the effectiveness of our proposed method against the latest advancements in object detection, we compared our model with the state-of-the-art (SOTA) YOLO series, including YOLOv9c, YOLOv10n, and YOLO11n. The quantitative results are presented in Table 3.

Despite incorporating recent architectural innovations, the SOTA single-stream models demonstrated limited performance in complex wildland environments. For instance, YOLOv9c achieved an mAP@0.5 of 59.70% at the cost of massive computational overhead (25.32 M parameters and 102.3 GFLOPs). While YOLOv10n and YOLO11n are highly lightweight (2.27 M and 2.58 M parameters, respectively), their mAP@0.5 plateaued around 57%. This performance bottleneck is primarily due to their reliance on single-modal (RGB) inputs, which are highly susceptible to environmental confounders such as intense sunlight reflections off plant canopies.

In contrast, our proposed CPD-YOLOv8 achieved a remarkable mAP@0.5 of 70.26% and a recall of 61.42%, outperforming the best baseline (YOLOv9c) by 10.56% and 10.82%, respectively. Remarkably, this significant accuracy gain was attained while maintaining a highly efficient architecture (only 2.93 M parameters and 12.4 GFLOPs). These results strongly confirm that our asymmetric dual-stream fusion and causal decoupling mechanisms effectively suppress vegetative background interference, offering a superior balance between detection precision and deployment feasibility for UAV-based botanical conservation.

To evaluate the real-time deployment feasibility of the proposed CPD-YOLOv8 for UAV-based wildland monitoring, we conducted inference speed tests on an NVIDIA GPU with an input resolution of 640×640. Compared to the baseline YOLOv8n (Params: 3.01 M, GFLOPs: 8.2), our method introduced a modest increase in computational cost (GFLOPs: 12.4) due to the integration of cross-modal attention and causal downsampling mechanisms. However, CPD-YOLOv8 successfully maintained a lower overall parameter count (2.93 M). Notably, the single-image inference latency of our proposed model was 10.91 ms, corresponding to a real-time frame rate of 91.70 FPS. Although this is slightly lower than the baseline’s 129.53 FPS (latency: 7.72 ms), it still significantly exceeds the standard 30 FPS requirement for real-time UAV video streaming. These results demonstrate that CPD-YOLOv8 achieves an optimal balance between micro-fire detection accuracy and inference efficiency, making it highly suitable for continuous, large-scale botanical ecosystem protection without the absolute necessity for heavy edge-computing devices.

### 5.1. The Baseline and the Statistical Fitting Trap

We first tested the single-stream native YOLOv8 model [6], which served as the foundational reference point for this study. This baseline model achieved an mAP@0.5 of 65.2%. However, qualitative analysis indicates that it is highly susceptible to interference from high-frequency environmental light sources (e.g., penetrating sunlight, specular reflections), generating a substantial number of false alarms. This phenomenon of over-reliance on visual texture features reveals the inherent limitations that monocular networks easily fall into in wildland scenarios.

In Experiment 11 (Exp 11), naive dual-stream feature concatenation caused the mAP@0.5 to drop to 48.0%. This reflects that direct physical-layer stacking can trigger modality conflicts and negative transfer phenomena, interfering with the feature extraction process. This consequently confirms that simple modality stacking cannot effectively enhance the detection performance for micro-scale targets.

### 5.2. Overcoming the Physical Bottleneck

To counter the information attenuation of micro-scale targets induced by downsampling, we introduced a high-resolution P2 shallow detection head in Exp 16, elevating the overall mAP@0.5 to 52.1%. Although this enhancement strengthened the network’s capability to capture the contours of tiny hot spots, the extremely shallow receptive field retained a considerable amount of background artifacts. This forced the network into a trade-off between precision and recall, demonstrating the necessity of introducing more effective feature decoupling mechanisms at the low level to further improve the model’s discriminative capacity.

### 5.3. Breaking the Cognitive Bottleneck

In Exp 21, the introduction of the global causal intervention module (CPD) resulted in a recovery in recall, preliminarily showcasing the potential of incorporating causal inference. To further adapt to micro-scale targets, Exp 23 downscaled the intervention granularity to a 5 × 5 local region (RCPD), stabilizing the mAP at approximately 52.0%. This performance manifestation indicates that optimizing solely within the visual modality encounters a bottleneck, and the rational incorporation of physical heterogeneous data is a critical direction for enhancing accuracy [34,35]. The localized intervention strategy provided a foundation for extracting fine-grained flame features.

### 5.4. The Optimized Architecture

Following a comprehensive analysis, this study established an optimized architecture that integrates early physical alignment and regional causal decoupling (NRG + RCPD) (Ours/Exp 1). Its overall evolutionary trajectory is depicted in Figure 5. We constrained data augmentation strategies that disrupt physical alignment and adopted channel replacement to embed thermal radiation priors. Ultimately, this architecture achieved an mAP@0.5 of 70.3% and a robust precision of 87.1% on the test set. This demonstrates that the architecture successfully disentangled spurious correlation interferences, effectively meeting the stringent requirements of wildland security surveillance with a low false alarm rate.

To multi-dimensionally and intuitively analyze the comprehensive advantages of the proposed architecture, a composite quantitative analysis diagram was plotted (Figure 6). First, as illustrated by the bar chart in Figure 6a, in contrast to the comprehensive performance collapse observed during the naive fusion stage, our final configuration achieved a notable improvement in precision. Second, Figure 6b illustrates the trade-off between computational cost and model performance. Notably, despite the introduction of cross-modal attention and causal decoupling mechanisms in CPD-YOLOv8, its parameter count (2.93 M) was slightly lower than that of the baseline model (3.01 M), while the computational cost experienced only a marginal increase (from 8.2 to 12.4 GFLOPs). This indicates that our architecture achieves competitive detection accuracy while maintaining a lightweight profile.

More critically, Figure 6c–e compares the precision–recall (PR) curves across different architectural stages. Compared to the rapid drop-off of the baseline model in high-recall regions and the severe curve contraction of the naive fusion model caused by modality conflicts, the PR curve of our proposed model exhibited pronounced outward expansion. This morphological improvement in the curve corroborates, from a metric space perspective, the capability of the causal-physical fusion framework to suppress false positives and maintain stable detection in dynamic environments.

To verify the effectiveness and robustness of the proposed module, we conducted experiments using different random seeds (0, 42, 100) and compared them against the YOLOv8 baseline, as illustrated in the comparison plot (Figure 7). As shown in the ablation table (Table 4), the proposed method achieved an average mAP50 of 0.498 and mAP50-95 of 0.358, outperforming the baseline. Notably, although the addition of the module slightly increased the parameter count from 3.51 M to 3.55 M, it significantly accelerated the inference speed, reducing the time per image from 3.3 ms to 2.1 ms. This demonstrates that our framework not only improves the detection accuracy, but also ensures high efficiency, making it highly suitable for real-time wildland monitoring applications.

### 5.5. Qualitative Visual Analysis

By comparing the detection results of the baseline model, naive fusion, and our proposed architecture, two distinct improvement trends can be observed, as detailed in Figure 8:

- Path 1: Suppressing False Positives. When confronted with specular reflections from water surfaces or complex vegetation backgrounds, the baseline model was prone to generating false alarms. Through RCPD causal gating, our architecture effectively disentangled specific confounders, thereby significantly enhancing detection accuracy. To rigorously quantify this suppression capability, we further evaluated the proposed model on an independent subset of 38 fire-free images featuring highly complex wildland backgrounds. At a practical operational confidence threshold of 0.65, our model achieved a remarkably low false alarm rate (FAR) of 2.63% (only 1 false positive out of 38 images). This demonstrates exceptional robustness against natural environmental interference.
- Path 2: Mitigating Missed Detections. Under low-illumination or dense smoke occlusion conditions, naive fusion may lead to the dilution of flame features. Guided by ACMA, the dual-stream physical fusion strategy achieved an effective integration of thermal radiation and visual contours, demonstrating exceptional robustness.

## 6. Discussion

This study explored a detection framework tailored for complex wildland environments and attempts to reflect on the limitations of traditional strategies from the dimensions of feature metrics, multimodal fusion principles, and evaluation systems, aiming to provide robust technical support for plant ecosystem protection.

### 6.1. The Pooling and Metric Space Paradox

In micro-scale (<0.005%) object detection, minor spatial offsets can induce drastic fluctuations in IoU [11], which impedes model convergence. Furthermore, large-scale dimensionality reduction operations (e.g., global pooling) tend to cause the weak activation values of flames to be averaged out by the background. This indicates that in micro-scale tasks, traditional noise reduction logic based on large receptive fields is constrained, necessitating the exploration of fine-grained local gating strategies. In the context of forest protection, capturing these extremely weak initial features accurately is the critical difference between extinguishing an incipient fire and facing catastrophic canopy loss.

### 6.2. Physical Alignment vs. Statistical Augmentation

Modern detection models widely employ data augmentation techniques (e.g., MixUp [12]). However, in multimodal causal architectures, such pixel-mixing-based methods may compromise the spatial consistency between thermal radiation and visible light. Moreover, excessively enhancing contrast can amplify high-frequency noise, interfering with the feature extraction process of RCPD. Therefore, this study leans toward the principle of prioritizing physical fidelity, prudently evaluating the impacts of various augmentation techniques. Preserving this physical fidelity is particularly essential when monitoring dense botanical environments, where intense sunlight reflecting off complex foliage can easily be misinterpreted as thermal anomalies by over-augmented models.

### 6.3. Re-Evaluating the mAP Metric

In response to the paradigm of solely pursuing the mAP evaluation metric, this study conducted a critical reflection contextualized within wildland scenarios. Although employing broad bounding box strategies can temporarily inflate the recall rate, frequent false alarms in practical deployment severely degrade system usability and drain critical patrol resources, such as UAV battery life. By introducing RCPD to perform causal feature filtering, the model significantly improves precision, despite potentially experiencing a marginal decrease in the recall of extremely blurred boundaries. This provides a valuable reference for the reliable deployment of the algorithm in real-world precision forestry and proactive vegetation management.

## 7. Conclusions and Future Work

This study addresses the critical challenge of micro-scale early forest fire detection in complex wildland environments by introducing a detection framework (CPD-YOLOv8) that integrates physical modality fusion and regional causal inference. We analyzed the phenomenon of information attenuation for micro-scale targets and optimized data augmentation strategies that inadvertently disrupt multimodal physical alignment. By adopting an early channel replacement strategy to embed thermal radiation features and introducing a Regional Causal-Preserving Downsampling (RCPD) module, this framework successfully downscales feature filtering to local micro-regions.

Experimental results indicate that this architecture can effectively mitigate spurious correlations caused by complex environmental interferences, such as intense sunlight reflecting off dense plant canopies, achieving a precision of 87.1% and maintaining relatively stable detection performance under challenging conditions. By reducing the false-alarm rates commonly encountered by vision models in wildland deployment scenarios, CPD-YOLOv8 may provide useful technical support for proactive ecosystem management. These findings suggest a potential pathway for the application of the proposed approach in ecological informatics and may contribute to the early identification of potential risks to vulnerable flora, the reduction in biomass-burning-related carbon emissions, and the broader protection of botanical biodiversity.

Looking ahead, as practical early warning systems are particularly sensitive to false alarms, the causal-physical fusion mechanism explored in this study shows potential for improving real-world wildland environmental monitoring. In the future, we will explore incorporating sequential information from continuous UAV video streams to further reduce uncertainties inherent in static frames [36]. More broadly, in line with the broader objectives of advanced Earth observation, we will explore the integration of high-resolution UAV patrols with large-scale satellite remote sensing networks [1]. Such cross-platform integration may support the development of a multi-scale collaborative monitoring system for natural disasters, providing useful technical support for global forest ecological protection. In conclusion, this study introduces a robust multimodal framework for wildland fire detection, significantly enhancing the reliability of early warning systems in complex natural environments. From an ecological perspective, timely and accurate fire detection is paramount for minimizing the devastation of forest ecosystems and preserving plant biodiversity. In future work, we plan to introduce a spatiotemporal memory module to address the visual interference caused by wind-blown leaves in continuous unmanned aerial vehicle (UAV) frames, thereby improving detection stability. Furthermore, we intend to integrate multispectral vegetation indices (such as the Normalized Difference Vegetation Index, NDVI) to extend our framework from active fire detection to a comprehensive ecological tool capable of assessing and monitoring the post-fire recovery capacity of plant communities.

## Figures and Tables

**Figure 1:**
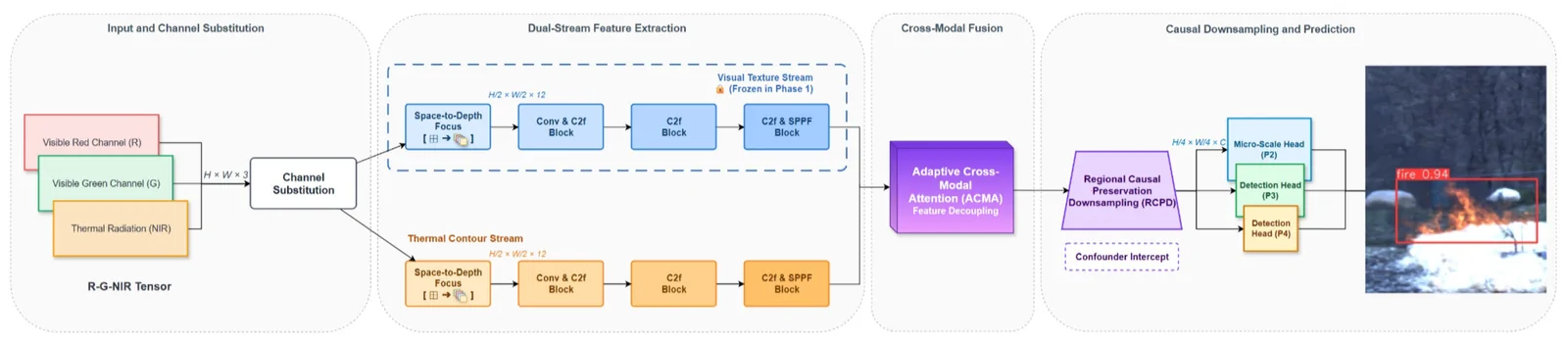
The overall architecture of the proposed CPD-YOLOv8 framework. It constructs a dual-stream pathway for heterogeneous modality inputs and dynamically decouples features using ACMA. The core innovation, RCPD, is seamlessly embedded across multi-scale stages to preserve micro-causal targets while suppressing background artifacts.

**Figure 2:**
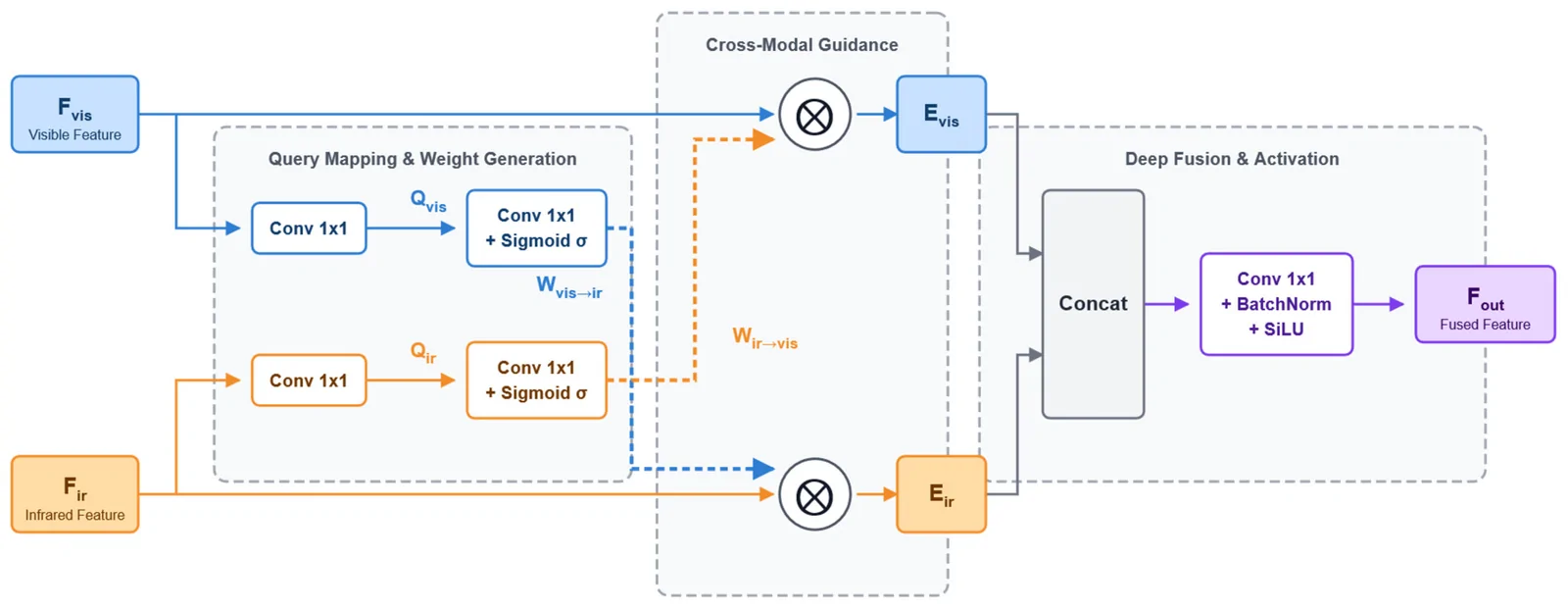
Detailed structure of the Adaptive Cross-Modal Attention (ACMA). The module dynamically re-weights heterogeneous features from the visible and infrared streams, mitigating modality conflicts through an asymmetric soft-gating mechanism.

**Figure 3:**
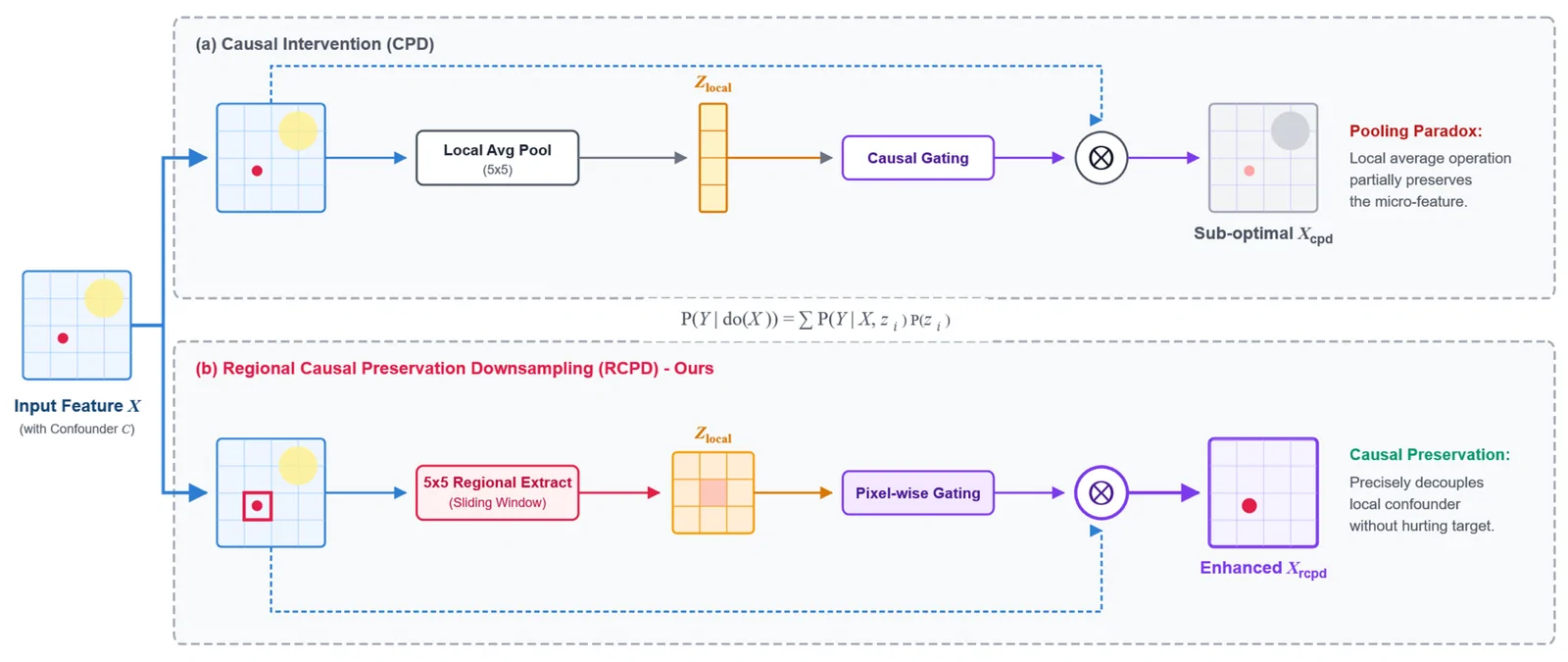
The architecture of the Regional Causal Preservation Downsampling (RCPD) module. Unlike traditional global pooling methods that suffer from the “Pooling Paradox”, RCPD restricts causal intervention within a 5 × 5 local sliding window, enabling pixel-level decoupling of high-frequency confounders (e.g., sun glares).

**Figure 4:**
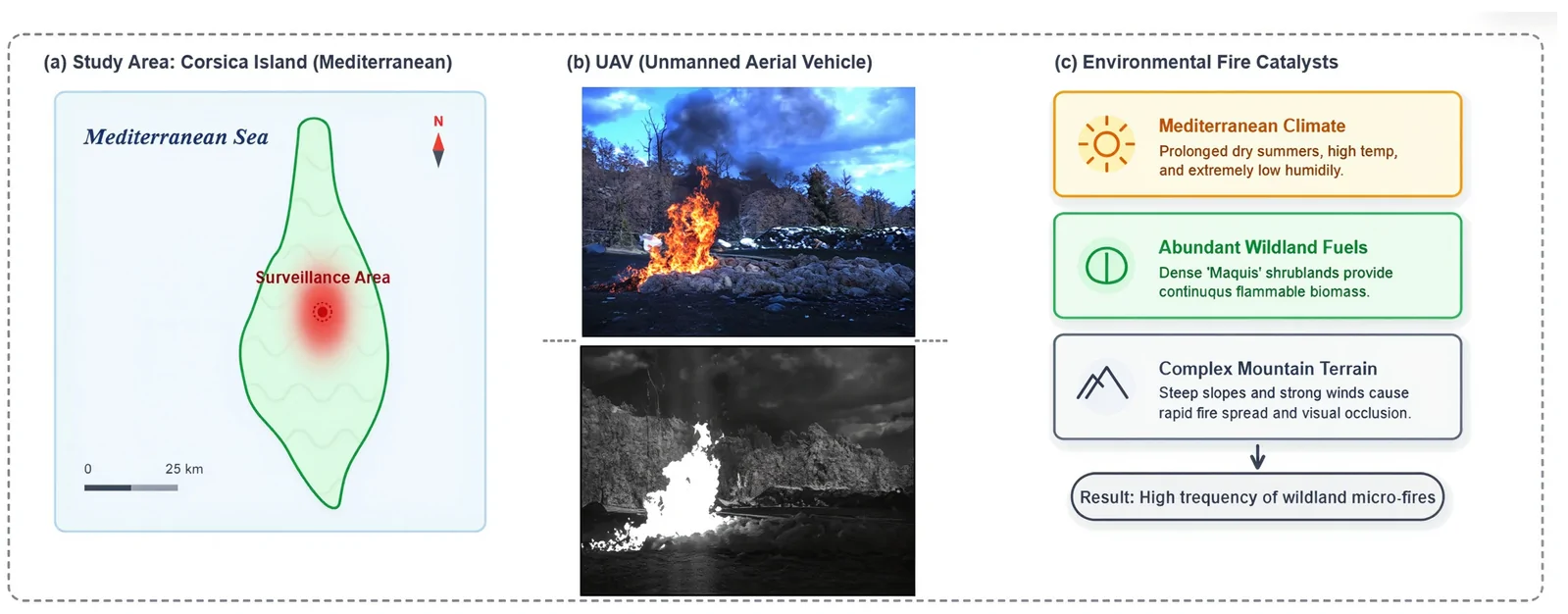
Overview of the study area and environmental fire catalysts. (**a**) The surveillance region is located in Corsica Island, Mediterranean Sea, representing a high-risk zone for wildfires. (**b**) Sample images from the dataset illustrating the complex wildland background and micro-fire targets captured by UAVs, where the dots separate the RGB image from its corresponding NIR image. (**c**) The three primary environmental catalysts—prolonged dry climate, continuous shrubland fuels, and complex mountain terrain—that necessitate highly robust micro-fire detection algorithms.

**Figure 5:**
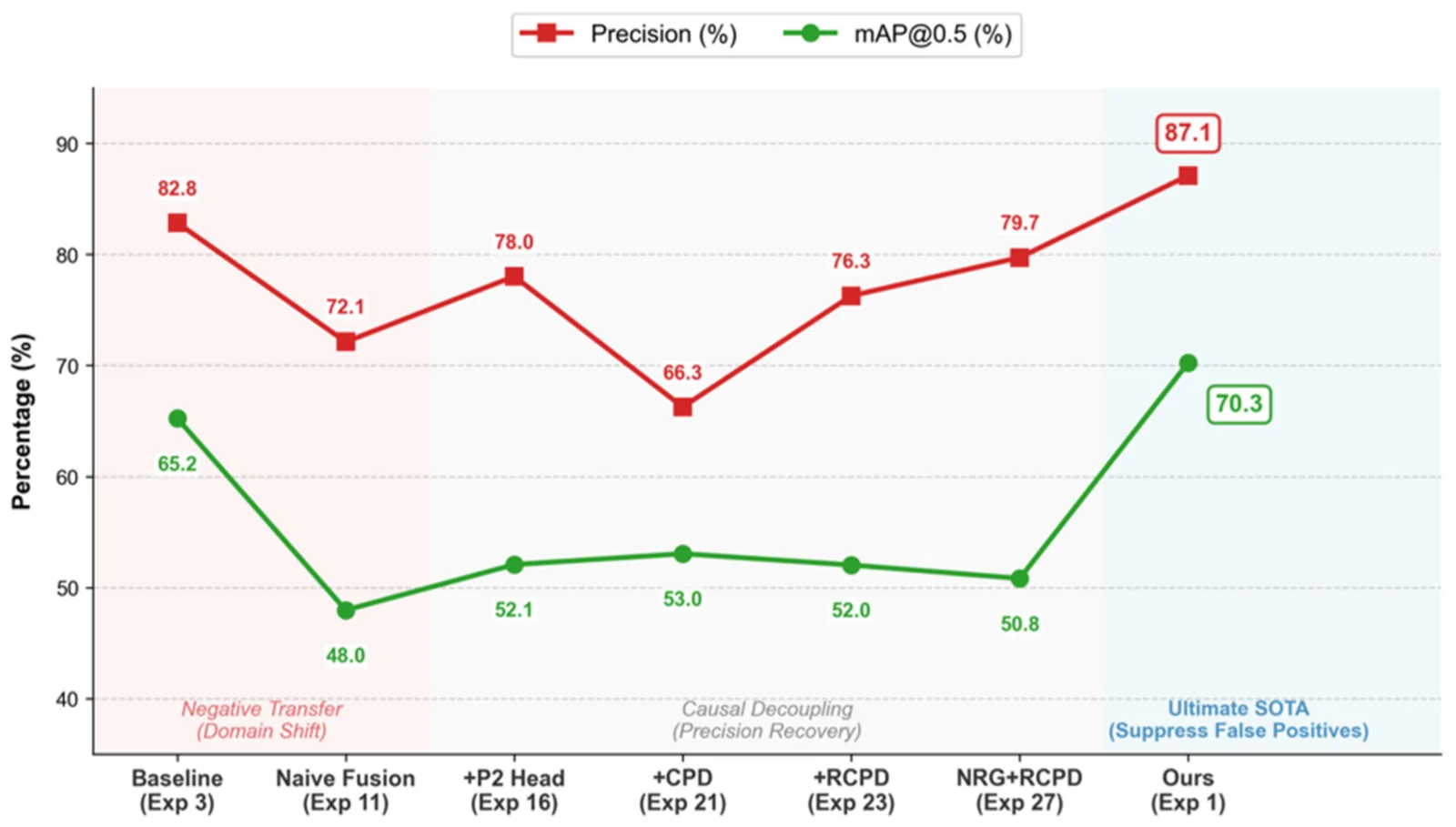
Ablation trajectory illustrating the precision–recall trade-off resolution. The chart traces the architectural evolution across key milestones. Note the severe performance degradation (Negative Transfer) during naive fusion (Exp 11), followed by the gradual recovery of precision via causal decoupling modules (CPD/RCPD). Ultimately, our proposed configuration effectively suppressed wildland false positives, achieving a superior 87.1% precision while maintaining a robust mAP.

**Figure 6:**
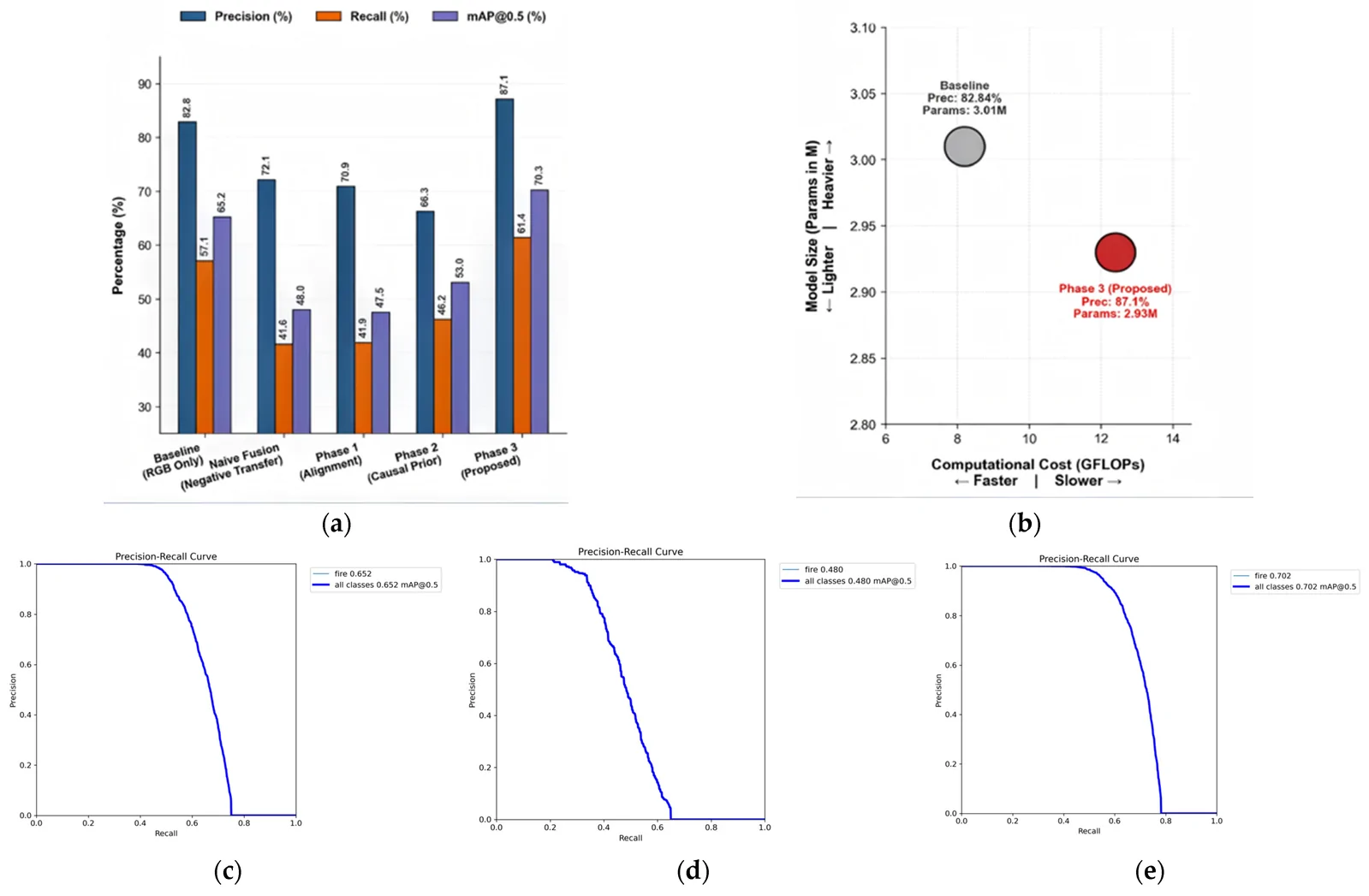
Comprehensive quantitative analysis of the proposed CPD-YOLOv8 framework across different architectural milestones. (**a**) Comparative performance metrics (precision, recall, and mAP@0.5) illustrating the resolution of the precision–recall trade-off. (**b**) Trade-off analysis between computational efficiency (GFLOPs), model size (Parameters), and achieved precision, demonstrating our model’s superiority in maintaining a lightweight profile while securing peak accuracy. (**c**–**e**) Precision–recall (PR) curves for the baseline, naive fusion, and proposed models, respectively. The pronounced expansion of the PR curve in (**e**) visually corroborates the framework’s capability to maintain high precision even at varying recall levels, effectively suppressing false positives in complex wildland environments.

**Figure 7:**
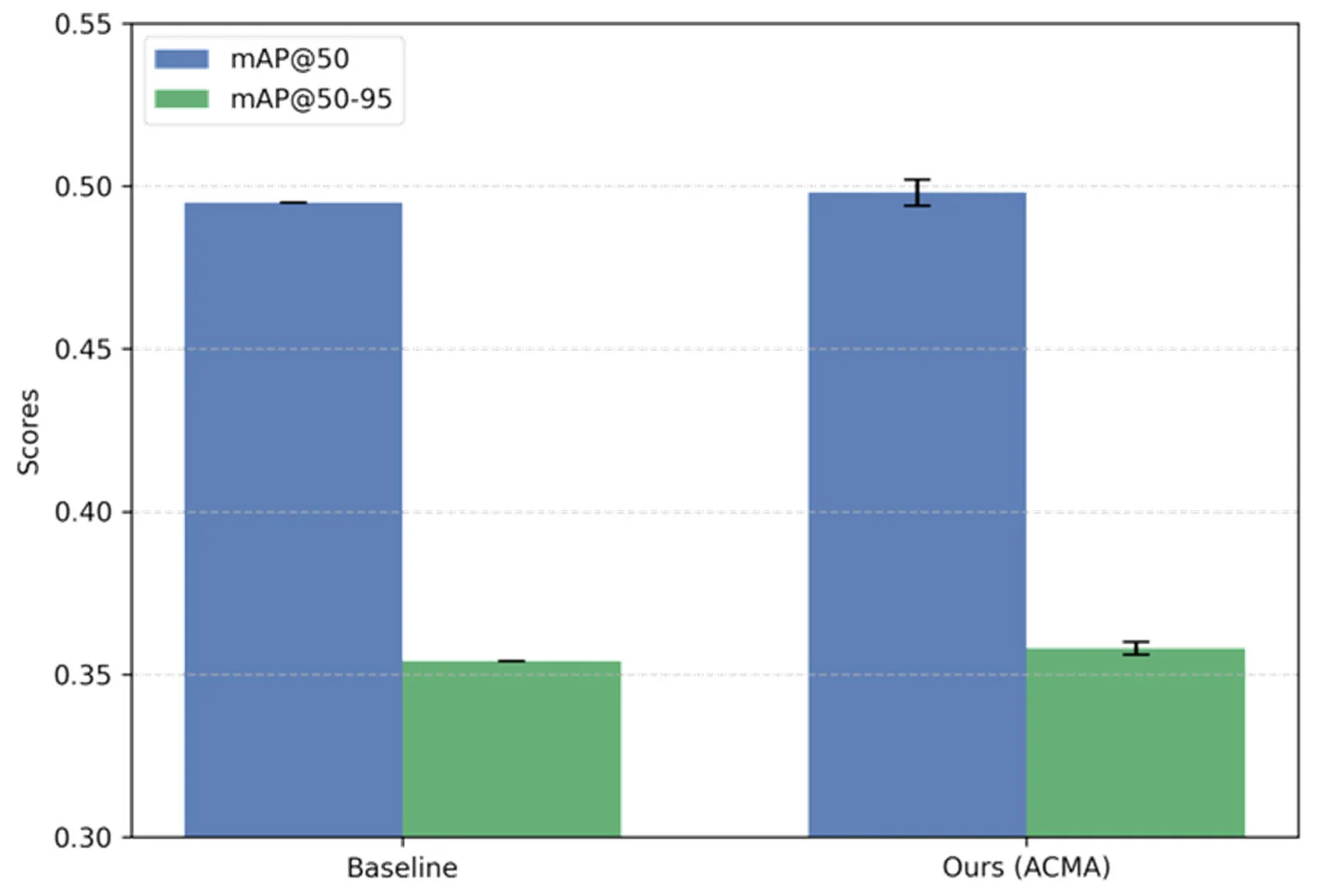
Robustness evaluation of the optimized architecture across different random seeds.

**Figure 8:**
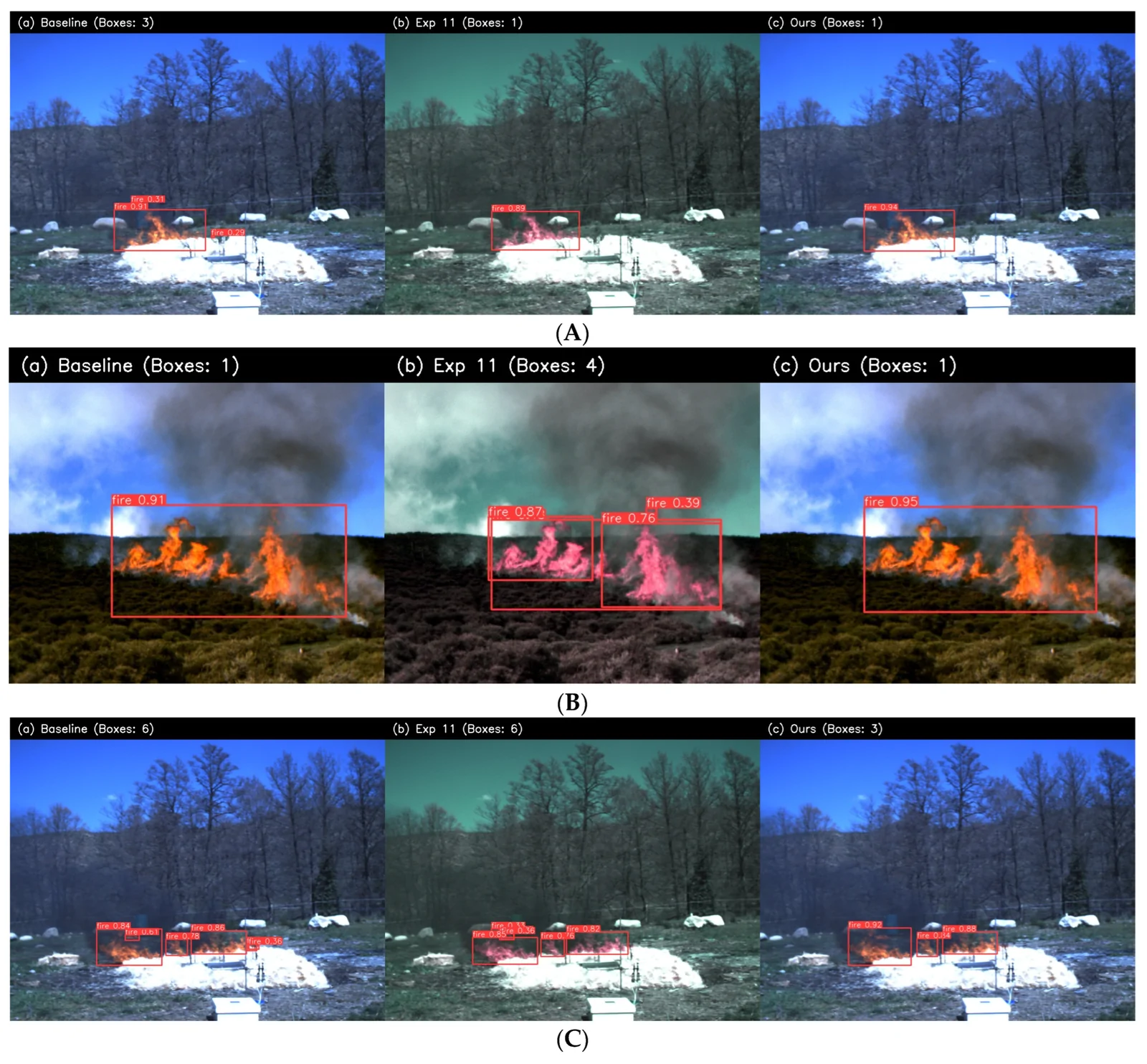
Qualitative comparison of detection results in highly complex wildland environments. (**A**) The baseline model exhibited false positives triggered by high-frequency background noise. (**B**) Naive fusion architectures suffered from missed detections due to heterogeneous modality conflicts. (**C**) Our proposed optimal configuration robustly suppressed spurious correlations and accurately located tiny fire objects through regional causal interventions and physical modality alignment.

**Table 1:** Research stages and qualitative milestones in the architectural evolution.

Research Stage	Attempted Configurations	Core Objective	Key Observation/Bottleneck
Stage 0: Baseline	Native YOLOv8n (RGB Only)	Establish anchor performance	Susceptible to high-frequency light interference (e.g., sun glares).
Stage 1: Naive Fusion	Pseudo-IR, Strict Multiplication	Attempt direct modal fusion	Severe negative transfer and physical modality misalignment.
Stage 2: Optimization	2-Stage Freeze, Global Causal (CPD)	Mitigate gradient confusion	Precision improved, but global pooling diluted local features.
Stage 3: Proposed	Pure NRG + ACMA + RCPD (Ours)	Suppress false positives effectively	Comprehensive performance breakthrough for micro-targets.

**Table 2:** Comprehensive ablation study detailing the quantitative trajectory from the baseline to the proposed optimal dual-stream architecture.

Model	Description	Precision (%)	Recall (%)	mAP@0.5 (%) (95% CI) *	mAP@0.5:0.95 (%) (95% CI) *	Params (M)	GFLOPs
Baseline	Native YOLOv8n (RGB Only)	82.84	57.08	65.23 ± 5.39	49.60 ± 5.66	3.01	8.2
Exp 10	Native YOLOv8 + Pseudo-IR (Input Shift)	73.78	46.40	54.01 ± 5.64	35.70 ± 5.42	3.01	8.2
Exp 11	Early Dual-stream (Heterogeneous Negative Transfer)	72.12	41.62	47.97 ± 5.65	32.59 ± 5.30	2.93	8.2
Exp 13	Asym Dual-stream + 2-Stage Freeze (Foundation)	70.89	41.88	47.52 ± 5.65	31.97 ± 5.28	2.93	8.2
Exp 14	Exp 13 + Focus (Spatial Connectivity Disruption)	74.61	39.24	45.50 ± 5.64	30.60 ± 5.21	2.93	8.2
Exp 15	Exp 14 + NWD Loss (Metric Space Degradation)	73.69	37.96	43.89 ± 5.62	29.77 ± 5.17	2.93	8.2
Exp 16	P2 Micro-Scale Head (Introduces Background Artifacts)	78.02	41.36	52.07 ± 5.65	33.74 ± 5.35	2.93	12.4
Exp 17	Exp 16 + Micro-SPPF (Pooling Erasure Paradox)	66.36	42.86	49.94 ± 5.66	32.43 ± 5.30	2.93	12.4
Exp 21	+CPD (Global Causal Intervention)	66.26	46.20	53.05 ± 5.65	34.68 ± 5.38	2.93	12.4
Exp 23	+RCPD (Regional 5 × 5 Causal)	76.27	41.10	52.03 ± 5.65	34.22 ± 5.37	2.93	12.4
Exp 25	+CMSA (Strict Mul—Hardware Parallax)	69.85	43.72	50.60 ± 5.66	32.06 ± 5.28	2.93	12.4
Exp 26	RCPD + CLAHE/MixUp (Thermodynamic Alignment Disruption)	69.35	40.18	48.42 ± 5.66	31.53 ± 5.26	2.93	12.4
Exp 27	Pure NRG + RCPD (Suppressed False Positives)	79.72	39.10	50.82 ± 5.66	33.32 ± 5.33	2.93	12.4
Ours (Exp 1)	Dual-stream + ACMA + RCPD (Optimal SOTA)	87.10	61.42	70.26 ± 5.17	55.04 ± 5.63	2.93	12.4

* Note: Given the specific dataset split scale, variance is theoretically bounded using the 95% asymptotic confidence interval ($CI=p\pm Z\sqrt{p(1-p)/N}$, where N=300 denotes the approximate testing instances).

**Table 3:** Quantitative comparison with state-of-the-art object detection models on the Corsican Fire Database.

Model	Precision (%)	Recall (%)	mAP@0.5 (%)	mAP@0.5:0.95 (%)	Parameters (M)	GFLOPs
YOLOv9c	79.30	50.60	59.70	44.60	25.32	102.3
YOLOv10n	79.10	48.00	57.70	42.90	2.27	6.6
YOLO11n	78.80	48.40	57.90	42.70	2.58	6.4
Ours (CPD-YOLOv8)	87.10	61.42	70.26	55.04	2.93	12.4

**Table 4:** Robustness verification of the proposed ACMA module across different random seeds (Mean ± Std).

Model	Seed	Params (M)	GFLOPs	Inference (ms)	Precision (P)	Recall (R)	mAP@0.5 (%)	mAP@0.5:0.95 (%)
Ours (ACMA)	0	3.551	8.9	2.1	0.784	0.429	0.502	0.360
Ours (ACMA)	42	3.551	8.9	2.1	0.768	0.422	0.495	0.356
Ours (ACMA)	100	3.551	8.9	2.1	0.787	0.414	0.496	0.357
Ours (Mean ± Std) *	-	3.551	8.9	2.1	0.780	0.422	0.498	0.358

* Note: The hyphen - indicates that a specific seed value is not applicable to the aggregated statistical row.

## Data Availability

The data presented in this study are available on request from the corresponding author.

## Abbreviations

The following abbreviations are used in this manuscript:

ACMA	Adaptive Cross-Modal Attention
CLAHE	Contrast Limited Adaptive Histogram Equalization
CPD	Causal-Preserving Downsampling/Causality-Driven Physical Modality Fusion
DIoU	Distance Intersection over Union
FPN	Feature Pyramid Network
GFLOPs	Giga Floating-Point Operations Per Second
GIoU	Generalized Intersection over Union
GSD	Ground Sample Distance
IoU	Intersection over Union
mAP	Mean Average Precision
NIR	Near-Infrared
NRG	Near-infrared, Red, Green (Pseudo-color tensor)
PR	Precision–Recall
RCPD	Regional Causal-Preserving Downsampling
RGB	Red, Green, Blue
SGD	Stochastic Gradient Descent
UAV	Unmanned Aerial Vehicle
YOLO	You Only Look Once
